# Cerebral Tumor

**Published:** 1883-02

**Authors:** 


### CEREBRAL TUMOR.

Prof. Hirschberg, the celebrated neurologist, publishes in the *Neurologisches Centralblatt* of December 15 a case of tuberculous tumor in the pons and in the choroid of both eyes. The patient, a little boy three years old, was very intelligent and of healthy parentage. Headache intense; vision normal; urine free from albumen and sugar. There were no motor disturbances in the trunk or limbs; but in the eyes was observed marked paresis of the left abducens and right rectus internus, the left rectus internus being only slightly affected. Vertical movements of the eyeball could be performed perfectly.

Wernecke states that the centre for lateral motion of the eyes lies in the lower portion of the pons*; it is bilateral, the left centre controlling lateral movements of *both* eyes to the *left*, the right governing similarly the right lateral motion of both. In this case the left centre wae involved, for, as Guddens has shown, the two abducens do not decussate. The process was only commencing in the right centre.

The opthalmascope at once decided the question as to the nature of the tumor. Besides recognition of its tubercular character, a classical "choked disc" of cerebral neoplasia was seen on both sides; the optic nerve was deep red in color and surrounded by a reddish-gray coating about one twenty-fifth of an inch thick. The veins were enormously dilated. Soon after headache increased and the patient became cross

---

* Archiv. f. Psych., 1877, and Centralblatt f. A., 1877, p. 189.

and somewhat somnolent. In a week there was marked weakness of the *right* lower limb, which was in keeping with the diagnosis, viz., that the *left* centre was mainly involved. Death occurred three months later, with profuse expectoration and incessant cough. Hirschberg states in conclusion that the disturbances of lateral movements were here of the highest diagnostic importance.

